# Preparation of *Aplysia* Sensory-motor Neuronal Cell Cultures

**DOI:** 10.3791/1355

**Published:** 2009-06-08

**Authors:** Yali Zhao, Dan O. Wang, Kelsey C. Martin

**Affiliations:** Dept. of Psychiatry and Biobehavioral Sciences, University of California, Los Angeles; Dept. of Biological Chemistry, University of California, Los Angeles; Semel Institute for Neuroscience and Human Behavior, University of California, Los Angeles

## Abstract

The nervous system of the marine mollusk *Aplysia californica* is relatively simple, consisting of approximately 20,000 neurons.  The neurons are large (up to 1 mm in diameter) and identifiable, with distinct sizes, shapes, positions and pigmentations, and the cell bodies are externally exposed in five paired ganglia distributed throughout the body of the animal.  These properties have allowed investigators to delineate the circuitry underlying specific behaviors in the animal^1^. The monosynaptic connection between sensory and motor neurons is a central component of the gill-withdrawal reflex in the animal, a simple defensive reflex in which the animal withdraws its gill in response to tactile stimulation of the siphon. This reflex undergoes forms of non-associative and associative learning, including sensitization, habituation and classical conditioning.  Of particular benefit to the study of synaptic plasticity, the sensory-motor synapse can be reconstituted in culture, where well-characterized stimuli elicit forms of plasticity that have direct correlates in the behavior of the animal^2,3^.  Specifically, application of serotonin produces a synaptic strengthening that, depending on the application protocol, lasts for minutes (short-term facilitation), hours (intermediate-term facilitation) or days (long-term facilitation).  In contrast, application of the peptide transmitter FMRFamide produces a synaptic weakening or depression that, depending on the application protocol, can last from minutes to days (long-term depression).  The large size of the neurons allows for repeated sharp electrode recording of synaptic strength over periods of days together with microinjection of expression vectors, siRNAs and other compounds to target specific signaling cascades and molecules and thereby identify the molecular and cell biological steps that underlie the changes in synaptic efficacy.

An additional advantage of the *Aplysia* culture system comes from the fact that the neurons demonstrate synapse-specificity in culture^4,5^.  Thus, sensory neurons do not form synapses with themselves (autapses) or with other sensory neurons, nor do they form synapses with non-target identified motor neurons in culture.  The varicosities, sites of synaptic contact between sensory and motor neurons, are large enough (2-7 microns in diameter) to allow synapse formation (as well as changes in synaptic morphology) with target motor neurons to be studied at the light microscopic level.

In this video, we demonstrate each step of preparing sensory-motor neuron cultures, including anesthetizing adult and juvenile *Aplysia*, dissecting their ganglia, protease digestion of the ganglia, removal of the connective tissue by microdissection, identification of both sensory and motor neurons and removal of each cell type by microdissection, plating of the motor neuron, addition of the sensory neuron and manipulation of the sensory neurite to form contact with the cultured motor neuron.

**Figure:**
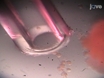


## Protocol

### Preparation (see solutions section at end of protocol for composition of solutions)

Prepare culture dishes. Coat glass well of Mattek glass-bottom culture dish with poly-l-lysine (made in sodium borate). Add enough to completely cover the glass well and leave for > 1hr (can be left overnight). Thoroughly remove the poly-l-lysine by rinsing in artificial seawater (ASW) 4-5 times. After removing the last rinse, add 2 mls of 50% L15 (supplemented with salts and containing L-glutamine at a final concentration of 2mM)/50% hemolymph. This culture medium must be in dish for at least one hour prior to plating cells so that the hemolymph coats the dish.
Clean the tools and Sylgard dishes with ethanol, rinse them thoroughly with ddH_2_0 and then place them for >1 hr under a UV light in the culture hood.
Prepare sharp electrodes for microdissection of neurons.****Using a microelectrode puller (e.g. Sutter Flaming Brown P-97), pull glass pipette electrodes with 1.5 mm outer diameter, 0.86 to 1.12 mm inner diameter and 100 mm length (e.g. AM system catalog #628000; World Precision Instruments TW150-4, 1.55/1.12). Use parameters that produce an electrode with a long and wispy fine tip of such high resistance that there is no capillary intake of fluid when placed in solution. The presence of a fluid meniscus means that the electrode tip will damage neuron during isolation. The Sutter electrode cookbook provides excellent guidelines for setting up electrode programs. We use a box filament, and a one-step pull to generate culture electrodes.
Prepare anesthetic solution (0.35M MgCl2), culture medium (L15 supplemented with salts and hemolymph), and protease solution (1% protease type IX, Sigma, 1 unit/mg, to a final concentration of 10 units/ml).
Animals: use adult 80-100 g *Aplysia* for isolation of sensory neurons and of LFS motor neurons. Use juvenile 1-4 g *Aplysia* for isolation of L7 motor neurons. Remove the animals gently from the seawater tanks, and maintain in seawater (in beaker, bucket or plastic bag) for no more than 30 minutes before beginning anesthesia and culture procedure.

### Culture procedure

### A. Culturing Pleural Sensory Neurons from 80-100 g *Aplysia*

Anesthetize the animals for removal of ganglia: Inject 0.35 M MgCl_2_ into adult *Aplysia* (80-100 g) using a 60 ml syringe with an 18 gauge 1.5 inch needle. Enter the foot of the animal at an angle of about 35 degrees, and do not enter too deeply. The goal is to inject into the hemocoel of the animal without penetrating the internal organs. The animal should become very distended and relaxed.
Dissect the ganglia: Pin the anesthetized animal on a dissecting dish, with a pin (18-gauge needles work well for 80-100 g animals) at the head and the tail, and the foot facing up. Hold the foot with a toothed forceps and cut through the skin and underlying connective tissue the full length of the foot (from head to tail) with a surgical scissors. Pin the two sides down. Using a forceps and scissors, cut the esophagus and pull to the side to expose the ganglia. Using a fine forceps and fine scissors, cut out the pleural pedal ganglia (the sensory neurons are in the pleural ganglia). Leave a fair amount of nerve since this makes it easier to pin down the ganglia after protease treatment.
Protease digestion of ganglia: Place the ganglia in protease solution (10 mg/ml of 1 unit/mg in L15) in an incubator set at 34.5°C (34-35°C is okay) for 2 hrs and 15min. Using the fine tip forceps to transfer and wash the ganglia in ASW three times. Keep the ganglia in L15. Note that the protease incubation time can vary. The purpose is to allow the connective tissue to be easily removed. If it is too difficult to desheath the ganglia without damaging the neurons, increase the protease incubation time. If it is extremely easy to desheath the ganglia, and the neurons are “soft,” reduce the protease incubation time.
Desheathing the ganglia: Transfer the protease-digested ganglia to a 60 mm or 35mm dish containing sylgard and L15. Viewing under a stereo-microscope (with external halogen illumination), remove pedal ganglia and pin down the pleural ganglia in the Sylgard dish in the proper orientation using insect pins and a fine forceps. With the fine forceps and a Vanna surgical scissors, carefully lift the connective tissue and cut it to expose the sensory cluster. Be careful not to ever touch or damage the neuronal somata. Remove any excess connective tissue from the dish and add more medium, making sure to never expose the desheathed ganglion to air.  The sensory cluster consists of approximately 200 clustered neurons of 40-50 micron diameter. Set a pin to make a “post” at a short distance from the ganglion (within the field of view).
Isolation of neurons: Using long, sharp glass culture electrode, pull out the identified neurons one by one by touching the cell body just off center (do not impale) and slowly and steadily pulling the cell soma, together with the axon, away from the ganglion. Gently tap against the pin to dislodge the neurons from the electrode.
Plating neurons: Use a pipetman (P10) to transfer individual neurons from the Sylgard dish to a culture dish. Before transferring the neurons, pipet culture medium into the tip so that the plastic tip is coated with hemolymph (this prevents the neurons from sticking to the plastic). Take great care to avoid exposing the neurons to any air bubbles or to any extreme forces (i.e. very gently take up and remove the neurons from the pipetteman). Distribute the neurons evenly throughout the dish. Use a sharp electrode to gently straight the processes and tap them down to the bottom.
Incubation: Leave the dishes on microscope stage at room temperature for no less than 3 hours. We routinely leave them overnight. Make sure that the microscope stage is unperturbed during this period. Cover the stage with aluminum foil to protect from light. Gently transfer them to an 18°C incubator.
Cultures should adhere to the dish within approximately 3 hrs and new neurite growth should be visible within approximately 6-12 hrs. The growth of isolated sensory neurons reaches a plateau by DIV 3 or 4. Note that isolated sensory neurons doe not form autapses or chemical synapses with one another, although they do form electrical gap junctions.

### B. Preparation of sensory neuron-motor neuron cocultures

Sensory neurons form synapse with target motor neurons in culture. The most commonly used motor neurons are the LFS motor neurons and the L7 motor neuron, from the abdominal ganglion. The LFS motor neurons are isolated from adult (80-100 g) *Aplysia*, and there are approximately 20 LFS motor neurons per abdominal ganglion. The L7 motor neuron is isolated from juvenile (1-4 g) animals, and there is only one L7 per abdominal ganglia. LFS motor neurons are 40-50 microns in diameter, are interspersed among the LE sensory neurons close to the root of siphon nerve on the ventral surface of the abdominal ganglia, and are characterized by a subtle dark pigment spot in each soma. The L7 motor neuron is 100-150 microns in diameter and is present on the dorsal surface of the ganglion, on the middle edge of the left side of the ganglion. While it is more economical to use LFS motor neurons, the large size of the L7 motor neuron is advantageous for some experiments. The L11 motor neuron, also on the dorsal left surface of the abdominal ganglion, caudal to L7, is a nontarget motor neuron and can be effectively used as a control with which the sensory neuron fasciculates but does not form chemical synapses.

Anesthetize the animals for removal of ganglia. For LFS motor neurons, do as described for pleural sensory neurons. For L7 motor neurons, inject 0.35 M MgCl_2_ into juvenile *Aplysia* (1-4 g) using a 10 ml syringe with a 21-gauge needle.
Dissect the abdominal ganglia: Pin the anesthetized animal on a dissecting dish as described above (using 21 gauge needles for juvenile animals). Using a fine forceps and fine scissors, cut out the abdominal ganglia.
Protease digestion of ganglia: This is done as described for pleural sensory neurons, except that the digestion time is reduced to 1 hr 45 min for ganglia from juvenile (1-4 g) animals.
Desheathing the ganglia: Transfer the protease-digested ganglia to a 35 or 60 mm dish containing sylgard and L15. Viewing under a stereo-microscope (with external halogen illumination), in the ganglia down in the Sylgard dish in the proper orientation using insect pins and a fine forceps. With the fine forceps and a Vanna surgical scissors, carefully lift the connective tissue and cut it to expose the neuronal cell bodies. To isolate the LFS motor neurons, pin the ganglion so that the ventral surface is facing up, and with a forceps pull the connective tissue sheath back to expose the entire half of the ganglion containing the LFS motor neurons (to your right), desheath the remaining parts of the ganglion, remove any connective tissue from the dish and add more L15. To isolate the L7 or L11 motor neuron, pin the ganglion so that the dorsal surface is facing up, and with a forceps pull the connective tissue sheath back to expose the half of the ganglion containing both L7 and L11 (to your left). Be careful not to ever touch or damage the neuronal somata. Place a pin to make a “post” at a short distance from the ganglion (within the field of view).
Isolation of neurons: Remove neurons with a sharp electrode as described for the sensory neurons. The LFS neurons are differentiated from neighboring LE sensory neurons on the basis of a small dark pigment spot in their somata. The L7 neuron can be differentiated from the L11 neuron first because the L11 is caudal to L7, because the L11 cell body is more oblong in shape while the L7 cell body is rounder and because the L11 axon tends to branch into two close to the cell body.
Plating neurons: Use a pipetman (P10) to transfer individual neurons from the Sylgard dish to a culture dish as described for the sensory neurons.
Pairing with sensory neurons: Let the motor neurons adhere to the culture dish for at least 1 hr. Then add the isolated sensory neurons with a pipetman, delivering each sensory neuron adjacent to a plated motor neuron. Using a sharp glass electrode carefully straighten out the sensory neuron axon, and coax the sensory cell body to a position next to the motor neuron, at a distance equal to or slightly less than the length of the sensory cell axon. Using the glass electrode, coax the sensory axon to come into contact with the motor neuron, very gently using the side of the tapered electrode to finally have the sensory axon physically contact the axon of the motor neuron. Do not lift the culture dish, but rather gently glide the dish along the microscope stage.
Leave the dishes on microscope stage at room temperature for no less than 3 hrs and transfer to an 18°C incubator as described for the sensory neurons.
Cultures should adhere to the dish within approximately 3 hrs and new neurite growth should be visible within approximately 6-12 hrs. Sensory neurons form glutamatergic synapses with motor neurons very quickly--as soon as the cell are adherent enough to perform sharp electrode recording (3-5 hrs), an excitatory post-synaptic potential can be recorded. Synaptic connectivity continues to increase until DIV 3, and is then stable until DIV7. The neurons should show elaborate neurite outgrowth during the first one to three days in culture.

### C. Preparation of Hemolymph

Hemolymph is used as a growth factor in *Aplysia* culture (analogous to the use of fetal calf serum in mammalian cell culture). It is collected from large (500 g-1 kg) animals, and the best time to collect hemolymph (anecdotally) is during the spring (mid-March to June). Swaddle the animal in a disposable underpad so that only a small portion of the animal is exposed, clean that portion of the skin with ethanol, and then hold it while another person uses a sterile razor blade to make an incision in the exposed area. Squeeze the animal so that the hemolymph squirts into a clean beaker, making sure that it does not contact the dirty skin of the animal. The hemolymph fills the hemocoel of the animal (i.e., the animal is basically a sac of hemolymph). Rewrap the animal once or twice, make a new incision and squeeze hard to collect as much hemolymph as possible (collect in a new beaker so that if it becomes contaminated, the first collection is still usable). Hemolymph from each animal should be kept separately (i.e., do not pool hemolymph from different animals). Spin the hemolymph at 2000 x g for 10 min to remove blood cells. Aliquot the supernatant in 10 ml aliquots, label by animal and store at -80°C. Note that hemolymph stored at -20°C forms a precipitate in medium. A new aliquot of hemolymph must be used every time culture medium is prepared, and the aliquot should be thawed just prior to preparing the medium. Do not refreeze after thawing.

## Discussion

The successful preparation of *Aplysia* sensory-motor cultures has a somewhat slow learning curve, since it involves the development of fine motor skills associated with microdissection and manipulation of individual neurons viewed through a stereo-microscope. In our experience, it takes approximately 1-3 weeks of practice to obtain healthy isolated sensory neurons in culture and an additional 1-3 weeks to learn how to pair sensory neurons with motor neurons. We routinely prepare cultures in a Labconco Clean Bench, although it is possible to prepare them on any laboratory bench or desk, as long as the microscope is unperturbed during the culturing (any vibrations or mechanical disturbances prevent the neurons from adhering). Since the neurons grow in seawater at 18°C, bacterial and fungal contaminations are significantly less common than in mammalian cell culture. The single most important variable in culture preparation is the quality of the animal. *Aplysia* can either be purchased from vendors who collect them directly from the Pacific Ocean (Alacrity, or Marinus), or from the University of Miami Mariculture facility, which collects breeding pairs from the Pacific and then grows *Aplysia* through one breeding cycle. As such, the animals are genetically heterogeneous, and, in the case of the animals collected from the ocean, they are also heterogenous in terms of their environmental history. There is some seasonality to the quality of neurons obtained from *Aplysia*, with the worst period usually occurring in August, and another lower quality period around December. Another important variable is the hemolymph. It is wise to test different batches of hemolymph for their growth-promoting capacity, and to use the same batch of hemolymph for a set of experiments.

Despite the difficulty of preparing *Aplysia* neuronal cultures, they possess a number of unique and valuable advantages to the study of synapse formation and synaptic plasticity. They form monosynaptic connections in culture that can be monitored by sharp electrode recording for periods of days. Well characterized protocols exist that elicit forms of plasticity that have clear parallels in the behavior of the animal. Stimuli can be applied to the bath or to subsets of synapses6,7, and the geometry of cultures can be varied so that one sensory neuron contacts a single motor neuron, or one sensory neuron with a bifurcated axon contacts two motor neurons, or two sensory neurons contact an individual motor neuron. Molecular and cell biological pathways can be altered in individual neurons by microinjection of DNA, RNA, siRNA and other reagents, and the effects on neuronal structure and function, synaptic transmission and plasticity can be monitored with temporal and spatial resolution. The NIH and Broad Institute are nearing completion of the sequencing of the *Aplysia* genome, which should
